# Short-Term Outcomes of Abdominal Surgeries Performed with Epidural Anaesthesia in Italian Calves

**DOI:** 10.3390/vetsci12050417

**Published:** 2025-04-28

**Authors:** Gessica Giusto, Claudio Bellino, Anna Cerullo, Marco Gandini

**Affiliations:** Department of Veterinary Sciences, University of Turin, 10095 Grugliasco, Italy; gessica.giusto@unito.it (G.G.); claudio.bellino@unito.it (C.B.); anna.cerullo@unito.it (A.C.)

**Keywords:** calf, laparotomy, abdominal surgery, epidural anaesthesia, local anaesthesia

## Abstract

Abdominal surgery is widely described for several diseases in cattle. While in adults surgical procedures are generally performed in standing or in sternal recumbency, lateral or dorsal recumbency is preferred in calves. General anaesthesia is associated with several side effects, and epidural anaesthesia (EA) is a valid alternative anaesthetic protocol for several surgical approaches. Previous studies have already been published about abdominal surgery in calves for several diseases, but they are not focused on short-term outcomes. Therefore, there is a need to report a case series for calves that have undergone exploratory laparotomy under EA. General anaesthesia is limited in cattle due to its association with several complications, such as ruminal stasis, regurgitation and tympany, and alternative protocols need to be used. The aim of this study is to describe the surgical features and short-term outcomes of elective and emergency laparotomies in calves under EA. In more complex or longer-lasting surgery, with a median duration of 80 min, intravenous administration of xylazine was necessary. Short-term survival (defined as survival until discharge from the hospital) rate was 90.1%. The EA was sufficient to ensure adequate analgesia in most surgeries. This type of anaesthesia is easy to administer, with limited side effects. The results of this study support surgeons in decision-making in the event of pathologies requiring abdominal surgery.

## Simple Summary

Several abdominal pathologies that require surgery have been described in calves. The clinical features can be variable and depend on the type of pathology, duration of symptoms and the possible presence of concomitant diseases. Among the diseases that most commonly require surgery, umbilical problems are the majority, but gastrointestinal or genitourinary pathologies can also be encountered. This study describes the short-term outcomes of a series of laparotomies performed on calves for umbilical, gastrointestinal or genitourinary diseases under epidural anaesthesia. This study is unique in that it evaluates short-term outcomes following abdominal surgery in calves using epidural anaesthesia alone or combined with minimal intravenous sedation, an area not extensively investigated in previous studies. This study demonstrated that epidural anaesthesia was sufficient to perform various surgical procedures in the calf abdomen, with a short-term survival of 90.1%. Intra- and post-operative complications were limited and, in all cases, not associated with the anaesthesiologic protocol used.

## 1. Introduction

Although less common than pneumonia or diarrhoea, surgical abdominal diseases can occur in calves and are associated with various clinical conditions, depending on the severity of the disease and the duration of symptoms [1]. One of the most common conditions in calves includes umbilical disorders [1,2], such as omphalitis, omphalophlebitis, patent urachus, and umbilical hernias. In newborn calves, congenital conditions such as colon and/or anal atresia may also be present, requiring timely surgical resolution. Additionally, several other gastrointestinal diseases can occur during the first months of life, making the gastrointestinal tract one of the most common reasons for abdominal surgery in calves [1].

General anaesthesia and recumbency are often avoided in adult cattle due to their association with several side effects, such as regurgitation, bloating, and muscle damage [3]. While abdominal surgeries in adults are commonly performed with the animal standing or in sternal recumbency, calves are typically positioned in lateral or dorsal recumbency [1]. In calves, recumbency is preferred due to the difficulty of maintaining a quadrupedal stance during the operation and because most pathologies require a median ventral laparotomy, such as omphalectomy, herniorrhaphy, or exploratory laparotomy for acute abdominal emergencies [1]. If recumbency is necessary, the animal will likely need to be sedated [4].

Several injectable anaesthesia protocols for cattle have been reported in the literature [5]. However, the use of injectable anaesthesia is less recommended for young or geriatric animals due to the potential side effects of the active ingredients on liver and kidney metabolism [6]. Inhalation anaesthesia could be a viable alternative, but it requires expensive and difficult-to-transport circular breathing systems. Additionally, another important consideration is that the active ingredients used in food-producing animals are subject to specific legal constraints and may sometimes be limited in availability. Therefore, epidural anaesthesia may represent a valuable alternative in these cases [6].

Administration of local anaesthetic agents via the epidural approach may result in severe ataxia and decubitus, which can be a disadvantage if the surgery requires the animal to remain standing or be properly restrained. However, this approach could provide a good compromise in calves [7]. Several studies have already been published regarding abdominal surgical approaches in calves for various diseases [1,8,9,10,11,12], focusing on differences in surgical techniques, clinical features, and outcomes. However, few studies have reported the short-term outcomes of calves undergoing abdominal surgery. Therefore, there is a need to present a case series on calves that underwent exploratory laparotomy under epidural anaesthesia. This study is unique in that it provides a detailed evaluation of short-term outcomes following abdominal surgery in calves performed under epidural anaesthesia alone or in combination with minimal intravenous sedation, in contrast to most previous studies that focused primarily on technical descriptions or outcomes under general anaesthesia. This study describes the use of epidural anaesthesia in calves undergoing a variety of abdominal surgical procedures. We hypothesised that epidural administration of xylazine and procaine, combined with an inverted V-block, would provide effective restrain and analgesia in calves undergoing abdominal surgery.

## 2. Materials and Methods

Records of calves referred to the Veterinary Teaching Hospital of the University of Turin from 2013 to 2023 and submitted to exploratory laparotomy for gastrointestinal, urogenital, and umbilical diseases were retrospectively analysed. All calves undergoing abdominal surgery under epidural anaesthesia between January 2013 and December 2023 were included in this study. No cases were excluded.

A complete clinical evaluation, including biochemical tests, blood count, and abdominal ultrasonography, was performed on all calves upon admission. Intramuscular amoxicillin and clavulanic acid (7 and 1.75 mg/kg) and flunixin meglumine (2.2 mg/kg) were administered prior to surgery. Epidural anaesthesia was performed on all patients. The skin over the S5-C1 and C1-C2 intervertebral spaces was clipped and aseptically prepared. An 18-gauge, 3.75 cm needle was inserted at a slight cranio-caudal angle to the skin surface and advanced between two adjacent vertebrae (S5-C1 or C1-C2) using the “hanging drop” technique [13]. A syringe was then attached to the needle, and the anaesthetic solution, consisting of xylazine (0.05 mg/kg) and 2% procaine (0.3 mL/kg), was slowly injected. The dosages of xylazine (0.05 mg/kg) and 2% procaine (0.3 mL/kg) were selected based on previously published protocols [1,3] and clinical experience in calves undergoing abdominal surgery.

Muscle tone in the hind limbs was lost, and the calves reached a sternal position after a mean of 2.1 min (range 1.5–3.5 min) following injection. Calves were placed in sternal recumbency for 10 min, and responses to sensory stimulation of the perineal region were assessed to monitor the effectiveness of analgesia, before calves were moved to the surgical table and placed in dorsal or lateral recumbency. The abdomen was surgically prepared, and a local block was performed to provide additional analgesia to the skin and fascia near the incision site [14]. An inverted V-block was used before a ventral midline laparotomy, and an inverted L-block was used for a flank laparotomy. Omphalectomy involved the surgical excision of the infected umbilical remnant and associated vasculature. Herniorrhaphy included closure of the abdominal wall defect. Atresia coli cases required resection of the affected segment and intestinal anastomosis.

Heart rate (HR) was recorded throughout the procedure. Spontaneous forelimb movements, head movements, vocalisation, and an increase in HR were the criteria for administering intravenous (IV) xylazine (0.05 mg/kg). The duration of surgery was also recorded. All patients were monitored during surgery for respiratory rate, heart rate, and body temperature. Once the procedure was completed, the animal was placed in sternal recumbency on a soft surface and recovered in a single box. Once the procedure was completed, the animal was placed in sternal recumbency on a soft surface and recovered in a single box. All calves received postoperative antimicrobial therapy, non-steroidal anti-inflammatory drugs, and daily wound assessments. Wounds were cleaned with antiseptics, and pain was assessed clinically.

Surgical findings, intraoperative complications, and short-term outcomes were documented. The short-term outcome was defined as survival until discharge from the hospital. Owners or referring veterinarians were contacted by telephone to collect information on the post-discharge course of the patients. Descriptive statistics were generated for age, weight, sex, and breed for the two groups. Normality was assessed using the Shapiro–Wilk test for continuous variables. Sex and breed were compared using the chi-squared test, while age and weight were compared using the Mann–Whitney test. Significance was set at *p* < 0.05.

## 3. Results

Seventy-one calves were included in the study. Thirty-nine were male and thirty-two were female, with median age of 12 days (range 1–180 days) and median weight of 63 kg (range 30–350 kg). Sixty-one were Piedmontese calves, nine were Friesian calves, and one was a crossbreed. Thirty-nine out of seventy-one (55%) cases had infection of umbilical structures, twenty-seven (38%) calves had gastrointestinal problems and five (7%) calves had genitourinary problems. Thirty-eight out of seventy-one cases (53.5%) were referred for acute abdominal emergencies. Only epidural anaesthesia with xylazine/procaine combination was used in 43 calves (61%). In 28 (39%) calves, aside the epidural anaesthesia, IV administration of xylazine (0.05 mg/kg) was required to complete the surgical procedure. Details related to surgical findings and anaesthesia protocol are reported in Table 1.

The median duration of interventions in which only epidural anaesthesia was used was 45 min (range: 10–90 min). The further administration of IV xylazine was required in surgical procedures of longer duration, with a median duration of 80 min (range: 40–120 min), or when calves started recovering from the effects of sedation given by epidural anaesthesia (increased HR, spontaneous movement of head and forelimbs or vocalisation). Intravenous administration of xylazine was, in all these cases, sufficient to recover a sedation status and to lower the HR to baseline values. No significant differences in sex (*p* = 0.65), breed (*p* = 0.87), age (*p* = 0.97) and weight (*p* = 0.49) were found between cases in which epidural anaesthesia only was used and cases in which IV xylazine was also administered. Three calves underwent intraoperative euthanasia, two due to poor prognosis because of severe peritonitis and one for excessive extension of intestinal pathology. All the remaining calves reached the standing position without difficulty after surgery. Sixty-eight calves were recovered uneventfully without intraoperative complications. Three calves with atresia coli underwent postoperative euthanasia for peritonitis and postoperative ileus and one calf with omphalitis died postoperatively for severe peritonitis. In total, four calves experienced postoperative complications: three were euthanized due to peritonitis and one died postoperatively from the same cause. Pain was managed postoperatively with flunixin meglumine (2.2 mg/kg, once daily). Sixty-five out of seventy-one calves were discharged alive from the hospital (90.1%) from 3 h to 4 days after surgery (median = 2 days). No complications were attributed to the anaesthetic technique.

## 4. Discussion

This study describes the use of epidural anaesthesia in calves undergoing a variety of abdominal surgical procedures.

Although the use of epidural anaesthesia has been described previously for numerous surgical procedures in cattle and other species [15,16,17], there are few studies about the use of epidural anaesthesia for abdominal surgery in calves focused on surgical outcome. Although previous studies reported that epidural anaesthesia did not always prevent response to visceral manipulation [17,18], it was sufficient in most cases that underwent abdominal surgery in the present study. In 28 cases, additional sedation with IV xylazine was needed to obtain full restraint of the animal, in long-lasting surgeries or in cases of complex procedures such as nephrectomy, enterectomy, resolution of bladder lacerations or urethral obstructions. The most frequent pathologies reported were umbilical and gastrointestinal diseases. Omphalitis was the most common surgical problem, followed by atresia coli and umbilical hernia. Insufficient environmental and umbilical hygiene were the most common factors responsible for the formation of umbilical remnant infections [19,20]. The aetiology of atresia coli is not well understood and is multifactorial. Both for the atresia of the colon and for the umbilical hernia, a genetic predisposition has been recognised in different breeds [1,21,22,23,24]. The atresia of the colon and anus needs a timely intervention. The results of this study confirm the possibility of performing this type of surgery under epidural anaesthesia, avoiding the side effects of intravenous anaesthesia. General anaesthesia has been associated with higher complication rates, such as bloat, regurgitation, and prolonged recovery times, especially in ruminants [4,5]. The short-term survival rate (90.1%) observed in our study under epidural anaesthesia compares favourably with reported outcomes using general anaesthesia.

The results of this study confirm the possibility of performing this type of surgery under epidural anaesthesia, avoiding the side effects of intravenous anaesthesia. Epidural anaesthesia in combination with local block was sufficient to contain the animal in dorsal or lateral recumbency and to ensure analgesia during the surgical procedure in most cases. This is in line with what was previously reported by Kamiloglu and colleagues in 2005 [7]. However, in the latter study, epidural anaesthesia was always associated with the intravenous administration of xylazine. In our study, xylazine was administered only in cases in which signs suggesting reduced sedation were noted, such as increased HR, spontaneous movement, or vocalisation. Xylazine is one of the most used sedatives in cattle, but had several side effects. This sedative can cause ruminal stasis, increased urine and saliva production, and may cause respiratory depression. Moreover, recumbency position can cause a worsening of these problems, with increased risk of aspiration and pneumonia [25]. Xylazine can also cause ataxia, prolonging the time before the animal can reach the quadrupedal station after surgery. Finally, in subjects with gastrointestinal problems, xylazine, which causes a reduction in intestinal motility and ruminal activity [7], could cause a further worsening of intestinal function. The goal is therefore to limit the time in which the animal is kept in the dorsal or lateral recumbency and find alternative strategies, such as epidural anaesthesia. In this study, intravenous xylazine, therefore, was administered only when necessary to reduce the above-mentioned side effects. In most cases epidural anaesthesia alone has allowed numerous surgical procedures to be performed, safely respecting animal welfare. The survival rate in our study was high (90.1%) and complications were limited and not associated with the anaesthesia protocol. Although epidural anaesthesia has been widely used in cattle, it was adopted especially for surgical procedures of the anus, vulva, perineum, caudal udder, and scrotum [26] that were performed generally in standing, sternal or lateral recumbency [4,26]. Caudal epidural administration of procaine and xylazine in combination provide sufficient analgesia for several surgical procedures, but it is rarely used for abdominal surgery [7]. Some previous studies have reported that epidural anaesthesia could not prevent responses to visceral manipulation and, therefore, may not be suitable for long or complex surgeries [17,18]. However, the use of epidural anaesthesia eventually associated with IV xylazine could limit many of the disadvantages of general anaesthesia in young animals, and in our study was sufficient for the performance of several abdominal surgeries in calves. This study confirmed that epidural anaesthesia has the advantage of being easy to perform, safe, and having a minimal risk of systemic side effects, as previously reported [8,25]. This study demonstrates that it is possible to perform these procedures even in the field, limiting the need for equipment. The risks of ataxia and injuries in the awakening phase, in this study, are limited by the fact that there are only young animals with reduced weight and therefore also of easy and safe containment. Although intravenous xylazine was used in response to signs of inadequate anaesthesia (e.g., increased HR, movement, vocalisation), we acknowledge that sedation alone may not provide sufficient analgesia. However, all surgeries were supported by local regional blocks (e.g., inverted V- or L-blocks), and signs of nociception were carefully monitored. The absence of formal pain scoring remains a limitation of the study.

This study had some limitations. The retrospective nature of the analysis limited the possibility to retrieve full data for all cases included. Furthermore, the number of cases considered is low and mainly considered Piedmontese and Friesian calves referred to a single centre. Studies on different breeds or including other centres could lead to different results. One further limitation is the absence of continuous oxygen saturation monitoring and formal pain scoring. These would have provided further insight into the effectiveness and safety of the anaesthetic protocol.

## 5. Conclusions

Surgical approaches for calves for abdominal disorders had a high success rate. Epidural anaesthesia is confirmed as an effective technique abdominal surgery in calves. Epidural anaesthesia represents a practical and effective alternative to general anaesthesia for abdominal surgery in calves. It provides adequate analgesia with minimal systemic effects, and may safely be implemented in both clinical and field conditions.

This type of anaesthesia ensures effective and sufficient analgesia in patients with surgical umbilical diseases. In more complex and long-lasting surgeries, epidural anaesthesia has been shown to be effective in combination with intravenous sedative administration. Future randomised trials comparing the use of epidural anaesthesia with other anaesthesiologic protocols could provide useful results for surgeons and referring veterinarians in choosing the ideal protocol based on the problems encountered.

## Figures and Tables

**Table 1:** Details related to surgical features and anaesthesiologic protocol used.

Disease		Surgical Procedure	N° of Animals	N° of Animals—Epidural Anaesthesia Only	N° of Animals—Epidural Anaesthesia + IV Xylazine
Umbilical structure	Omphalitis	Omphalectomy	22	17	4
	Patent urachus		4	4	0
	Omphalophlebitis		1	1	0
	Urachus infection		3	2	2
	Umbilical hernias	Herniorrhaphy	9	8	1
Gastro- intestinal	Atresia coli	Intestinal anastomosis	12	1	11
	Atresia ani		3	3	0
	Atresia ani and atresia coli		1	0	1
	Mesenteric abscess	Intestinal reposition	1	0	1
	Jejunal volvulus		3	2	1
	Colon intussusception		1	1	0
		Colon bypass	1	1	0
	Peritonitis	Abdominal lavage	3	3	0
	Jejunal atresia	Side-to-side anastomosis	1	0	1
	Colon foreign body	Colon enterotomy	1	0	1
Urogenital	Urethral obstructions	Urethrostomy	2	0	2
	Bladder laceration	Cystoplasty	2	0	2
	Renal disease	Nephrectomy	1	0	1
Total			71	43	28

## Data Availability

The data presented in this study are available on request from the corresponding author.

## References

[1] Mulon P.Y., Desrochers A. (2005). Surgical abdomen of the calf. Vet. Clin. N. Am. Food Anim. Pract..

[2] Steiner A., Lejeune B. (2009). Ultrasonographic assessment of umbilical disorders. Vet. Clin. N. Am. Food Anim. Pract..

[3] Skarda R.T. (1996). Local and regional anaesthesia in ruminants and swine. Vet. Clin. N. Am. Food Anim. Pract..

[4] Nichols S., Fecteau G. (2018). Surgical Management of Abomasal and Small Intestinal Disease. Vet. Clin. N. Am. Food Anim. Pract..

[5] Greene S.A. (2003). Protocols for anaesthesia of cattle. Vet. Clin. N. Am. Food Anim. Pract..

[6] Rush J., Stockler J., Lin H.C., Passler T., Clark-Price S. (2022). Local and Regional Anesthesia in Food Animal. Farm Animal Anesthesia.

[7] Kamiloglu A., Kamiloglu N.N., Ozturk S., Atalan G., Kılıc E. (2005). Clinical assessment of epidural analgesia induced by xylazine-lidocaine combination accompanied by xylazine sedation in calves. Ir. Vet. J..

[8] Sutradhar B.C., Hossain M.F., Das B.C., Kim G., Hossain M.A. (2009). Comparison between open and closed methods of herniorrhaphy in calves affected with umbilical hernia. J. Vet. Sci..

[9] Abutarbush S.M., Naylor J.M. (2006). Obstruction of the small intestine by a trichobezoar in cattle: 15 cases (1992–2002). J. Am. Vet. Med. Assoc..

[10] Van Metre D.C., Callan R.J., Holt T.N., Garry F.B. (2005). Abdominal emergencies in cattle. Vet. Clin. N. Am. Food Anim. Pract..

[11] Roussel A.J., Cohen N.D., Hooper R.N. (2000). Abomasal displacement and volvulus in beef cattle: 19 cases (1988–1998). J. Am. Vet. Med. Assoc..

[12] Anderson D.E., Constable P.D., St Jean G., Hull B.L. (1993). Small-intestinal volvulus in cattle: 35 cases (1967–1992). J. Am. Vet. Med. Assoc..

[13] Anderson D.E., Edmondson M.A. (2013). Prevention and management of surgical pain in cattle. Vet. Clin. N. Am. Food Anim. Pract..

[14] Edmondson M.A. (2008). Local and regional anaesthesia in cattle. Vet. Clin. N. Am. Food Anim. Pract..

[15] Ko J.C., Althouse G.C., Hopkins S.M., Jackson L.L., Evans L.E., Smith R.P. (1989). Effects of epidural administration of xylazine or lidocaine on bovine uterine motility and perineal analgesia. Theriogenology.

[16] LeBlanc P.H., Caron J.P., Patterson J.S., Brown M., Matta M.A. (1988). Epidural injection of xylazine for perineal analgesia in horses. J. Am. Vet. Med. Assoc..

[17] Trim C.M. (1989). Epidural analgesia with 0.75% bupivacaine for laparotomy in goats. J. Am. Vet. Med. Assoc..

[18] Lewis C.A., Constable P.D., Huhn J.C., Morin D.E. (1999). Sedation with xylazine and lumbosacral epidural administration of lidocaine and xylazine for umbilical surgery in calves. J. Am. Vet. Med. Assoc..

[19] Marchionatti E., Nichols S., Babkine M., Fecteau G., Francoz D., Lardé H., Desrochers A. (2016). Surgical Management of Omphalophlebitis and Long Term Outcome in Calves: 39 Cases (2008–2013). Vet. Surg..

[20] Salci E.S.O., Salci H. (2012). Anatomo-physiological involution of umbilical cord, umbilical hygiene and etiopathogenesis of the umbilical lesions in calves. Res. Opin. Anim. Vet. Sci..

[21] Hayes H.M. (1974). Congenital umbilical and inguinal hernias in cattle, horses, swine, dogs and cats: Risk by breed and sex among hospital patients. Am. J. Vet. Res..

[22] Priester W.A., Glass A.G., Waggoner N.S. (1970). Congenital defects in domesticated animals: General considerations. Am. J. Vet. Res..

[23] Syed M., Shanks R.D. (1992). Atresia coli inherited in Holstein cattle. J. Dairy Sci..

[24] Syed M., Shanks R.D. (1992). Incidence of atresia coli and relationships among the affected calves born in one herd of Holstein cattle. J. Dairy Sci..

[25] Weaver A.D., Atkinson O., Jean G.S., Steiner A., Weaver A.D., Atkinson O., Jean G.S., Steiner A. (2018). General Considerations and Anesthesia. Bovine Surgery and Lameness.

[26] Ismail Z.B. (2016). Epidural analgesia in cattle, buffalo, and camels. Vet. World.

